# Carminic Acid Linked to Silica Nanoparticles as Pigment/Antioxidant Bifunctional Excipient for Pharmaceutical Emulsions

**DOI:** 10.3390/pharmaceutics12040376

**Published:** 2020-04-19

**Authors:** Francisco Arriagada, Catalina Ugarte, Germán Günther, María Angélica Larraín, Víctor Guarnizo-Herrero, Santi Nonell, Javier Morales

**Affiliations:** 1 Instituto de Farmacia, Facultad de Ciencias, Universidad Austral de Chile, 5110033 Valdivia, Chile; francisco.arriagada@uach.cl; 2 Facultad de Ciencias Químicas y Farmacéuticas, Universidad de Chile, 8380494 Santiago, Chile; caugarte35@hotmail.com (C.U.); ggunther@ciq.uchile.cl (G.G.); mlarrain@uchile.cl (M.A.L.); 3 Facultad de Farmacia, Universidad Complutense de Madrid, 28040 Madrid, Spain; victor08@ucm.es; 4 Institut Químic de Sarrià (IQS), Universidad Ramon Llull. Via Augusta 390, 08017 Barcelona, Spain; santi.nonell@iqs.url.edu

**Keywords:** carminic acid, emulsion, antioxidant, silica nanoparticles, nanoexcipients

## Abstract

The incorporation of pigments and natural polyphenols into inorganic matrices, resulting in a hybrid material that improves the resistance and chemical stability of the pigments and the antioxidant capacity of the materials, has been of great interest to the pharmaceutical, chemical and food industries. The aim of this work was to prepare and characterize a bifunctional pigment–antioxidant nanomaterial-based carminic acid-decorated solid core-mesoporous shell silica nanoparticles, evaluating its properties as a pigment, its antioxidant capacity and its properties as a chemical stabilizer of emulsions. The chemical stability of oil-in-water (O/W) Pickering emulsions was evaluated determining the stability of vitamin E solubilized in the oil phase. Carminic acid was attached through the action of coupling ethylcarbodiimide hydrochloride (EDC)/*N*-hydroxysuccinimide (NHS) agents, and the resulting spherical and homogeneous nanoparticles showed a diameter close to 175 nm. A notorious change of emulsion color was observed by the addition of the nanomaterial. Emulsions showed an attractive pink color, and when the pH was adjusted to pH 3 and pH 9, a change in color was observed, analogous to carminic acid in solution. The nanomaterial incorporation also improved chemical stability, decreasing vitamin E consumption to 9.26% of the initial value, demonstrating an important antioxidant effect of the developed nanomaterial.

## 1. Introduction

In pharmaceutical, cosmetic and food products, as emulsions, color is a crucial factor defining quality and consumer perception. Emulsion coloration depends on several droplet properties (size, concentration, aggregation and relative refractive index), and when present, also on pigment absorption efficiency (mainly determined by its absorption spectra and concentration). Thus, the emulsion’s visual appearance results from the combination of scattered and absorbed light [1]. Carminic acid (CA) is a common natural hydroxyanthraquinone pigment, used for coloring pharmaceutical, cosmetic or food formulations in a wide range of red shades [2], dependent on pH. In addition, it has excellent heat and light stability [3,4]. Moreover, besides these properties, several studies have reported CA to act as antioxidant against free radicals and reactive oxygen species (ROS), such as singlet oxygen [5,6,7,8]. 

Emulsions are thermodynamically unstable systems, which, by the addition of surfactants and/or solid particles or a mixture of surfactant with other amphiphilic polymers, can be kinetically stabilized [9,10,11,12]. When solid particles are used to stabilize an emulsion, it is known as a Pickering emulsion [13,14]. The “emulsifier-free” character of Pickering emulsions makes them attractive in applications where surfactants have detrimental effects on both human health and environment [15,16,17,18]. The enhanced stability of Pickering emulsions, with respect to classical emulsions stabilized by emulsifiers, is a supplementary advantage. The coating of droplets by solid particles constitutes a physical rigid barrier acting against coalescence; therefore, emulsions are efficiently stabilized [19,20].

Several materials have been used to prepare Pickering emulsions [21,22,23,24,25]; among them, silica is the most popular, because its surface is easily modified, either by chemical reaction or by physical adsorption of various compounds such as salts, low molecular weight surfactants, polymers and pigments and natural polyphenols, among others [25,26,27,28,29,30,31,32]. In particular, different types of silica materials (MCM-41, SBA-16, etc.) have been used for various pharmaceutical purposes, such as drug delivery systems (DDS) [33,34,35], to improve the dissolution rate of drugs [36], to limit cutaneous penetration of organic UV filters [37] or even as pigment (or drug) removers from water [38]. These porous materials present a high specific surface area that allows a large amount of drug to be incorporated; nonetheless, several authors have reported modifications to the synthesis process, in order to obtain improvements in the properties of silica nanoparticles. For example, the synthesis of core–shell silica materials (dense core and mesoporous shell) allows for a suitable density of particles for the adsorption at the interface of an oil-in-water (O/W) emulsion and, at the same time, to have a high surface area to incorporate different molecules of pharmaceutical interest. Among these different biomolecules, various studies have shown that adsorption and linkage of natural polyphenols on the silica surface maintains the antioxidant capacity, improving the stability of nanomaterial [39,40,41,42]. Norton et al. showed that silica particles could increase the oxidative stability of O/W emulsions by acting as a physical barrier between pro-oxidants present in a continuous phase and hydroperoxides located at droplet interface [17,43]. 

The incorporation of a pigment to inorganic matrices to obtain a hybrid material that improves the resistance and chemical stability of the pigments has been widely studied in several fields of application and different production methodologies [44,45,46,47,48].

The immobilization of a pigment onto silica nanoparticles is an attractive approach to obtain hybrid materials that take advantage of the carminic acid color and antioxidant properties, as well as the silica nanoparticle stable matrix. In particular, its incorporation into emulsions in the field of the pharmaceutical industry is of great interest. In skin applications and dermatological formulations, the size of nanoparticles is a crucial factor [49]. Some authors suggest that these kinds of nanoparticles are able to penetrate skin. For example, Rancan et al. demonstrated that 42 nm silica nanoparticles (SNPs), applied on human skin explants, with a partially disrupted stratum corneum, were localized in hair follicles and can be internalized by epidermal and dendritic cells. Other researchers have obtained similar conclusions [50,51]. On the other hand, some authors reported that in vivo, skin penetration of 55 nm diameter (111 nm hydrodynamic diameter in ultrapure water) functionalized silica nanoparticles was not observed after one or five consecutive days of topical application [52]. In addition, it is relatively well accepted that bigger size nanoparticles (75 nm and larger) cannot penetrate the skin [53]. In this work, we prepared nanoparticles of about 200 nm, which cannot penetrate the skin. The silica nanoparticles with carminic acid covalently linked could be a safe, efficient and novel bifunctional pigment–antioxidant nano-excipient to stabilize O/W pharmaceutical emulsions.

The aim of this work was to prepare and characterize carminic acid decorated solid core-mesoporous shell silica nanoparticles, evaluating their properties as a pigment, their antioxidant capacity and the chemical stabilizing properties of Pickering emulsions. Unlike physical adsorption, the chemical immobilization of polyphenols on the surface of solid core-mesoporous shell silica nanoparticles prevents their release by desorption and also prevents the formation of dimers and other aggregates that usually decrease their antioxidant capacity. The chemical stability of O/W Pickering emulsions was evaluated determining the stability of vitamin E (α-tocopherol) solubilized in the oil phase. This bioactive compound was selected as a model hydrophobic drug, because of its high susceptibility to oxidation.

## 2. Materials and Methods 

### 2.1. Materials

Carminic acid (CA, ≥92%) was purchased from Merck (Darmstadt, Germany). N-hydroxysuccinimide (NHS, 98%), *N*-(3-dimethylaminopropyl)-*N*’-ethylcarbodiimide hydrochloride (EDC, ≥98%), tetraethyl orthosilicate (TEOS, 98%), (3-aminopropyl)triethoxysilane (APTES, ≥98%), hexadecyltrimethylammonium bromide (CTAB, ≥98%), deuterium oxide (D_2_O, 99.990 atom % D), alfa tocopherol (Vitamin E, 96 wt. %), 3Å molecular sieves, and phenalenone were purchased from Sigma-Aldrich (St. Louis, MO, USA). Surfactants Tween 60^®^ and Span 60^®^ were obtained from Sigma-Aldrich, while glycerin, castor oil, mineral oil and cetyl alcohol were purchased from CRODA International PLC (CRODA Chile, Santiago, Chile). Ethanol (HPLC grade), acetonitrile (HPLC grade), methanol (HPLC grade), acetone (HPLC grade) and triethylamine (TEA, ≥99%) were obtained from Merck. Ammonium hydroxide (NH_4_OH, acs reagent 28%–30%) was purchased from J.T. Baker (Phillipsburg, NJ, USA). Deionized water (Milli-Q, 18.2 MΩ.cm) was used in all experiments performed. All materials were used as received.

### 2.2. Preparation of Silica Nanoparticles (SNPs)

SNPs were obtained by a modified Stöber method following slight modifications [54,55,56]. Briefly, for a typical synthesis of SNPs around 100 nm in diameter, ethanol (44 mL), deionized water (3 mL) and ammonium hydroxide (1.3 mL) were added to a round-bottomed flask and stirred for 10 min. Later, TEOS (1.73 mL) was added to the mixture and allowed to react for 3 h under stirring (850 rpm) at room temperature. The nanoparticles (SNPs) were obtained by centrifugation (15,000× *g*) and washed with water and ethanol several times. Finally, the product was dried and stored until further use.

### 2.3. Preparation of Solid Core-Mesoporous Shell Silica Nanoparticles (CSSNPs)

First, 2.3 g of CTAB and 15 μL of TEA were mixed in water, then 300 mg of SNPs, were added to the solution and stirred at room temperature for 30 min. The suspension was heated up to 80 °C and 150 μL of TEOS were added dropwise and stirred for 1 h at 80 °C. Then, the product was washed once with water, re-suspended in a 1 wt % solution of NaCl and stirred overnight to remove CTAB. The CTAB extraction process was carried out several times to ensure its complete removal. Finally, the product was obtained by centrifugation, dried and designated as CSSNPs.

### 2.4. Preparation of Amino-Functionalized Solid Core-Mesoporous Shell Silica Nanoparticles (ACSSNPs)

300 mg of the above CSSNPs were suspended in 50 mL of ethanol at 40 °C and 250 μL of APTES were added. The mixture was stirred for 12 h at 40 °C, and the modified nanoparticles were then centrifuged and washed with ethanol. The resulting nanoparticles (ACSSNPs) were dried and stored until further use.

### 2.5. Conjugation of Carminic Acid to Amino-Functionalized Core–Shell Silica Nanoparticles by EDC/NHS Coupling Chemistry (ACSSNPs-CA)

Grafting of carminic acid (CA) to ACSSNPs was achieved by coupling its -COOH group to the -NH_2_ group of ACSSNPs using the EDC/NHS coupling agents [57]. In a typical synthesis, 6.0 mg of CA, 4.6 mg of EDC and 5.6 mg of NHS were dissolved in water, and the mixture was sonicated for 15 min. After this, the mixture was added dropwise to a suspension of 100 mg of ACSSNPs, and the reaction was allowed to proceed under stirring for 6 h at room temperature [42]. The product was collected by centrifugation and washed three times with ethanol to remove by-products and unreacted reagents. The nanoparticles (ACSSNPs-CA) were dried and stored until further use.

### 2.6. Characterization of Nanoparticles

The hydrodynamic diameter of nanoparticles suspended in ethanol was measured by dynamic light scattering (DLS) at 25 °C, using a Malvern Zetasizer Nano ZS90 (Malvern, UK) with a detection angle of 173° and equilibration time of 120 s. Each measurement was performed three times. Zeta potentials were obtained using the same equipment at 25 °C with the nanoparticles suspended in deionized water. Morphological analysis using STEM was taken on an FEI^TM^ inspect F50 model microscope, with an accelerating voltage of 10.00 kV. Fourier transform infrared (FT–IR) spectra were obtained using an Interspec 200-X FT-IR spectrometer with 4 cm^−1^ resolution, between in 4000 and 400 cm^−1^, averaging 16 scans. The thermogravimetric analysis (TGA) were performed using a Netzsch TG 209 F1, under air flow of 20 mL min^−1^ with a heating rate of 10 °C min^−1^ in the range of 20–800 °C.

### 2.7. Determination of Carminic Acid Amount Covalently Linked to ACSSNPs

Determination of CA grafting onto nanoparticles was also quantified by a previously validated HPLC indirect method. Chromatographic analysis was performed on a Prominence HPLC system (Shimadzu, Tokyo, Japan) using a Chromolith Performance RP-18 monolithic (4.6 × 100 mm) from Merck. Isocratic elution was performed using a mobile phase consisting of methanol: 0.1% trifluoroacetic acid solution (30:70 *v/v*), at a flow rate of 1.0 mL/min. The sample injection volume was 50 µL, the run time was 14 min and the retention time was 9.0 min. PDA detector was set at 499 nm. Data were obtained from triplicate analysis.

### 2.8. Antioxidant Assay: Singlet Oxygen Quenching

Direct detection of singlet oxygen (^1^O_2_) near-infrared phosphorescence at 1275 nm was performed using a customized PicoQuant Fluotime 200 lifetime system [58]. A diode-pumped pulsed Nd:YAG laser (CryLas, FTSS355-Q, Berlin, Germany) working at 1 kHz repetition rate at 532 nm was used for excitation. The time-resolved ^1^O_2_ emission data were fitted to the Equation (1) [59] using GraphPad Prism 5 software:

(1) $$S\left(t\right)=S_{0}\frac{\tau_{\Delta }}{\tau_{\Delta }-\tau_{T}}\left(e^{-\frac{t}{\tau_{\Delta }}}-e^{-\frac{t}{\tau_{T}}}\right)$$

where S(t) is the ^1^O_2_ signal intensity at time t, S_0_ is an empirical parameter proportional to singlet oxygen quantum yield, τ_Δ_ is the ^1^O_2_ lifetime and τ_T_ is the triplet-state lifetime [59]. The total quenching rate constant (*k*_T_) for the deactivation of ^1^O_2_ by ACSSNPs-CA or free CA in acetone and deuterium oxide (D_2_O) was obtained by measuring the first-order luminescence decay of singlet oxygen in the presence and absence of quencher and using phenalenone as sensitizer. A control using CA-free nanoparticles under the same conditions above mentioned was measured. The *k*_T_ values were calculated from the slope of the Stern–Volmer plots, according to Equation (2):

(2) $$\tau^{-1}=\tau_{0}^{-1}+k_{q}\left[Q\right]$$

where τ^−1^ and τ_0_^−1^ are the singlet oxygen lifetime in presence and absence of quencher [Q], respectively [59].

### 2.9. Color Measurement

Color space values of ACSSNPs-CA dispersed in hydroxyethylcellulose solutions were determined using a Hunterlab (10D65) colorimeter (Reston, VA, USA), where color has a unique location, as defined by its Cartesian coordinates with respect to a* (red–green axis), b* (yellow–blue axis) and L* (lightness axis) for the brightness (CIE system) [60]. In addition, the color difference (ΔE) was calculated using coordinate geometry, considering that the human eye can perceive a color change corresponding to a value of ΔE > 1.5 [61].

### 2.10. Stability of Nanoparticles under Oxidative Conditions

Samples of 150 mg of ACSSNPs-CA dispersed in 7 mL of water were mixed with 3 mL of 30% H_2_O_2_ solution to obtain a final concentration of 15 mg mL^−1^. The mixture was homogenized in a vortex mixer. The samples were stored in vials, during 1, 2, 3, 4 and 5 days at 40 ± 2 °C and 70% RH ± 5%. After this, each sample was collected by centrifugation (15,000× *g*), washed with water and ethanol several times, and finally dried and stored until further use.

### 2.11. UV-Visible Spectroscopy Measurements

A single-beam UV-visible Agilent 8453 spectrophotometer (Agilent Technologies, Shanghai, China) equipped with 1 cm quartz cells was used to characterize the UV-visible absorption properties of CA solutions and ACSSNPs-CA dispersions in ethanol, deionized water, glycerin solutions and mineral oil and acetone using the same concentration. All absorption spectra were analyzed using UV-visible ChemStation Software of Agilent Technologies. The UV-visible absorption spectra for ACSSNPs-CA were performed for each suspension using ACSSNPs as blank, in order to subtract the scattering generated by the nanoparticles.

### 2.12. Incorporation of Nanoparticles in Emulsions

#### 2.12.1. Preparation of Emulsions

A total of 100 g of emulsion was prepared by mixing the aqueous phase (4.0 g glycerin and 78 g water) and the oil phase (7 g mineral oil, 1.5 cetyl alcohol, 3.0 g castor oil and 0.5 g vitamin E under vigorous stirring (1000 rpm) using an IKA Eurostar 20 Digital overhead stirrer during 5 min at 80 °C [62]. The surfactants (2.4 g Tween 60 and 1.1 g Span 60) were added in the oil phase. The preparation was cooled to room temperature under slow stirring. Water lost by evaporation was replenished. Three formulations were prepared, one incorporating ACSSNPs-CA, a second one with ACSSNPs, and a final emulsion without nanoparticles. To this aim, the nanoparticle powder (ACSSNPs-CA or ACSSNPs) was dispersed in glycerin and sonicated for 15 min. Then, 300 μL of water were added to the mixture. Finally, the emulsion was added in small quantities under vigorous stirring. Final concentration of nanoparticles in the emulsions were 0.1 wt %, 0.5 wt % and 1 wt %. All emulsions contain vitamin E 0.5 wt %, to simulate the composition of existing commercial products. The nanoparticle-free emulsions were designated as E-0. On the other hand, emulsions containing ACSSNPs and ACSSNPs-CA were designed as E-NP and E-NP-CA, respectively.

#### 2.12.2. Stability and Characterization of Emulsions

All emulsions were monitored visually at room temperature and at 40 °C to detect physical changes as phase separation or other instabilities for 120 h, to ensure a suitable and stable emulsion for all further experiments. The viscosity of samples was determined using an HB Brookfield viscometer DV2T, at 200 rpm and with needle No. 5. The form and size of the oil droplets was determined using an optical microscope. The emulsion color was evaluated by visual inspection [63,64].

In order to evaluate the antioxidant capacity of ACSSNPs-CA in the emulsion under stress conditions, vitamin E was used as lipophilic model in the dispersed phase. Vitamin E consumption was determined using an HPLC method previously developed and validated [65]. To perform the analysis, 25 mg of emulsion was mixed with 10 mL of isopropanol in a 50 mL volumetric flask. After this, the mixture was sonicated and diluted to required concentration with methanol. Subsequently, the sample was homogenized, filtered and measured by HPLC. The analysis was performed in a C-18 column with isocratic elution (methanol-water 97:3), pumped at a flow rate of 1.5 mL/min and the injection volume was 50 μL. PDA detector was set at 290 nm. The emulsion color was evaluated by visual inspection.

### 2.13. Statistical Analysis

Results are expressed as means ± standard deviation (SD) of a least three experiments using GraphPad Prism software version 6.01 (La Jolla, CA, USA). 

## 3. Results

### 3.1. Preparation and Characterization of ACSSNPs-CA

**Figure 1:**
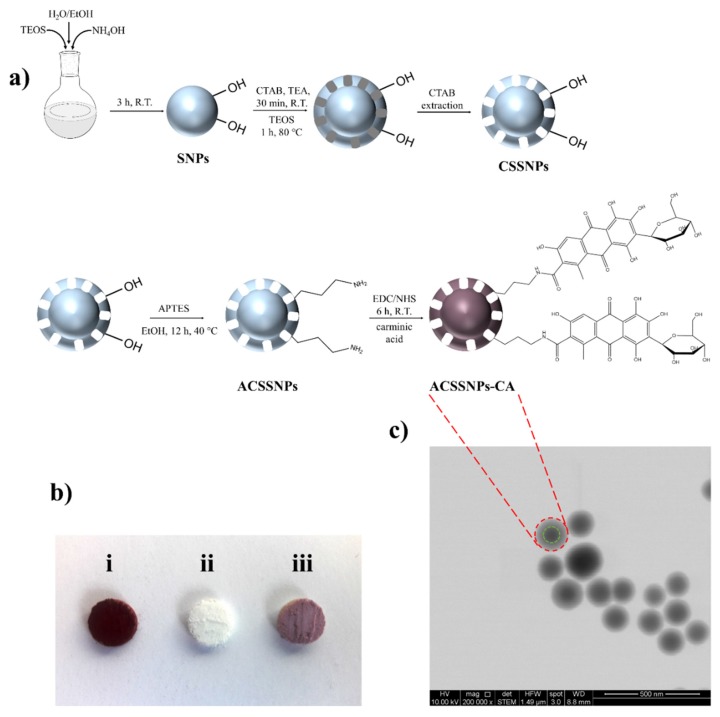
(**a**) Schematic illustration of the obtainment of amino-functionalized mesoporous core-shell silica nanoparticles with immobilized carminic acid (ACSSNPs-CA). (**b**) Powders of (i) carminic acid, (ii) ACSSNPs and (iii) ACSSNPs-CA. (**c**) TEM image of ACSSNs-CA.

A bifunctional (pigment and antioxidant) nanomaterial, based on silica superficially modified with carminic acid, was obtained. Initially, a uniform dense silica core with a diameter of 108 nm was prepared, which was then coated with a mesoporous silica using CTAB. After this, the surfactant was removed and nanoparticles with a hydrodynamic diameter of 162 nm were obtained (CSSNPs). The CSSNPs were amino-functionalized (with APTES, ACSSNPs) and then, carminic acid was immobilized onto the shell (Figure 1a). The porous shell thickness that allows the carminic acid linkage determined by TEM was 40 nm. To activate the -COOH group of carminic acid, EDC/NHS cross-linking agent were used, which facilitates the reaction with the -NH_2_ group on the surface of the amino-functionalized nanoparticles, yielding an amide bond. The resulting spherical and homogeneous ACSSNPs-CA showed a diameter of about 175 nm, determined by TEM images (Figure 1c) and DLS measurements. The zeta potential average of CSSNPs and ACSSNPs were −33.5 mV and +2.8 mV, respectively. The difference is due to the successful modification of the OH groups of the porous shell when functionalized with amino groups. After immobilizing the carminic acid (CA) onto ACSSNPs, the zeta potential of the obtained nanosystem (ACSSNPs-CA) decreases to −22.6 mV, showing a successful functionalization. TGA and HPLC results revealed that per each 100 mg of nanoparticles, 4.24 mg and 4.35 mg of carminic acid were incorporated, respectively. An average value of 4.30 mg was considered for all the experiments carried out.

Carminic acid linked to the nanoparticle (white powder) surface, confers them a purple color less intense than the characteristic red color of the carminic acid (Figure 1b). The presence of an amide bond was confirmed by FTIR. Typical of CSSNPs, the characteristic bands of (≡Si–O–Si≡) groups appear at 1200–1000 cm^−1^; in addition, bands at 2982 cm^−1^ and 2940 cm^−1^ that correspond to the stretching vibration of -CH_2_ present in the APTES propyl chain and the characteristic bending bands of NH_2_ group at 1518 cm^−1^ were observed for ACSSNPs. Furthermore, bands observed at 3300 cm^−1^ can be assigned to the stretching of the OH bond (adsorbed water). On the other hand, the ACSSNPs-CA spectrum additionally shows bands at 1638 cm^−1^ and 1544 cm^−1^ which correspond to amide-bond presence [66,67]. However, the best evidence of the successful conjugation of carminic acid was obtained when the nanomaterial was dispersed in solvents such as water, alkalized water (pH 12), acidified water (pH 1) and ethanol. Subsequently, the samples were stirred at room temperature for six hours, centrifuged and then the supernatant analyzed by HPLC. The results did not show the presence of desorbed CA, unlike the control samples, consisting of CA adsorbed onto ACSSNPs, in which the CA was completely desorbed. 

### 3.2. Antioxidant Capacity: Singlet Oxygen Quenching by ACSSNPs-CA

Many pharmaceutical products are constantly exposed to degradation agents. For example, traces of transition metals such as copper or iron are presented in various pharmaceutical products and can produce free radicals by redox reactions with organic or inorganic substrates, molecular oxygen or hydroperoxides [68]. In particular, products such as emulsions applied to the skin are commonly exposed to UVA and UVB radiation, which generates harmful effects on the skin as well as on the applied products [69]. It is known that UVA radiation produces deleterious effects, since different chromophores in the skin or sensitizing drugs are able to generate several reactive oxygen species (ROS) [70,71,72], in which singlet oxygen plays a major role [73,74]. Therefore, studying the interaction between singlet oxygen and ACSSNPs-CA (CA covalently linked) is of great interest due to the potential deactivation effect of a ROS, which gives this hybrid nanosystem the functionality not only as an emulsion stabilizer and a pigment, but also as a product with antioxidant activity.

The amount of singlet oxygen monitored as infrared luminescence signal, decreased in the presence of free carminic acid and in the presence of ACSSNPs-CA. The sum of the physical quenching rate constant (k_Q_) and chemical reaction rate constant (k_r_) corresponds to the total quenching rate constant (k_T_).

In acetone, the estimated value of k_T_ for free CA was 6.35 × 10^7^ M^−1^s^−1^ indicates that CA is able to quench singlet oxygen efficiently. The CA attached onto silica nanoparticles shows an enhanced singlet oxygen quenching (k_T_ = 1.30 × 10^8^ M^−1^s^−1^) compared to the free CA, which is completely protonated under the experimental conditions. 

In D_2_0, the total quenching rate constants (k_T_) for CA and ACSSNPs-CA were 1.46 × 10^7^ M^−1^s^−1^ and 1.67 × 10^8^ M^−1^s^−1^ respectively, similar to those obtained in acetone.

This could be explained due to the partial ionization of CA in the obtained nanosystem, favored by the free amino groups on the nanoparticles that produce a CA phenolate form, which is capable to quench ^1^O_2_ more efficiently than protonated CA. In addition, this is evidenced by a change of color from orange–red to intense purple, a characteristic color change when a violet tri-anionic molecule appears [75]. Another explanation could be the quenching effect of the silica matrix [76]; nevertheless, previously we reported that the effect in this nanosystem was not significant [42]. These results display a suitable antioxidant agent, the k_T_ value of this nanomaterial is similar or even greater than the value reported for a few recognized antioxidants such as quercetin or morin [77,78].

### 3.3. Nanomaterial: Color Evaluation and Relation with Its Antioxidant Capacity

To establish a direct correlation between color and remaining antioxidant capacity, a colorimetric determination was performed to ACSSNPs-CA under oxidative stress (oxidation with hydrogen peroxide) at 40 °C ± 2 °C and 70% RH ± 5% for 5 days. At initial time, when ACSSNPs-CA is non oxidized, the red component (a* 22.67 ± 0.18) is greater than the yellow component (b* 18.03 ± 0.14). Both colorimetric parameters decrease linearly when the nanomaterial is oxidized with hydrogen peroxide for 1 to 5 days at 40 ± 2 °C and 70% RH ± 5%. After five days of undergoing highly oxidizing conditions, the NPs decreases its red color by 50% (a* 11.95 ± 0.10), increasing its brightness (L*) from 17.96 ± 0.14 at initial time to 31.22 ± 0.25 at five days. The color differences calculated (ΔE) were between 2.46 ± 0.02 and 7.70 ± 0.06, greater than the limit (1.5).

**Figure 2:**
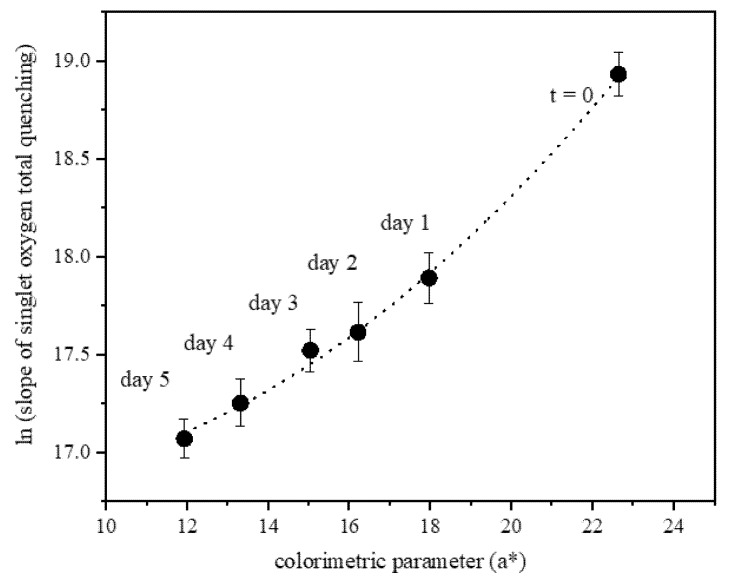
Relation between the colorimetric parameter (a*) versus the antioxidant capacity of ACSSNPs-CA after oxidation with H_2_O_2_ at 40 °C ± 2 °C and 70% RH ± 5% for five days.

Figure 2 shows the relation between the colorimetric parameter (a*) versus the antioxidant capacity of ACSSNPs-CA after oxidation with H_2_O_2_ at 40 °C ± 2 °C and 70% RH ± 5% for five days. The antioxidant capacity was determined as the slope of the Stern–Volmer curve for the total quenching of singlet oxygen. The results show an exponential relation between both factors, where in addition to the obvious loss of color, the antioxidant capacity also decreases. However, the antioxidant capacity after five days under stress conditions is still significant (2.60 × 10^7^ M^−1^s^−1^).

### 3.4. ACSSNPs-CA Incorporation in O/W Emulsion: Stability of Vitamin E

For oil-in-water (O/W) emulsions stabilized with surfactant; we used Tween 60 and Span 60, corresponding to a 30% of the oil phase. The selection of these surfactants is based on their known ability to stabilize O/W pharmaceutical emulsions. The pH of all the emulsions was adjusted to pH 5 using citric acid, considering that Binks et al. reported that the emulsification at neutral or alkaline pH stabilized with nanoparticles did not produce a stable emulsion [79,80]. 

The stability of the emulsions was investigated under stress conditions (oxidation and temperature). Emulsions prepared with and without vitamin E and with and without nanoparticles were stored at 40 °C for 24, 48 and 120 h and were periodically analyzed. 

The use of core-shell silica nanoparticles provides two great advantages. First, the solid core provides the required density to the nanoparticle to remain at the O/W interface. Second, the mesoporous shell allows to immobilize more carminic acid on the surface, compared to a non-porous particle. Additionally, we carried out the incorporation of carminic acid onto silica nanoparticles, as a control sample, by physical adsorption using the impregnation method. However, the loaded amount of carminic acid adsorbed was less than that obtained by the immobilization technique (data not shown); therefore, no quantitative experiments were performed with this material. 

A good physical stability of all emulsions was observed by optical microscopy analysis, confirming that the size of the drops has not changed (average diameter 20 μm), even after 120 h of storage at 40 °C. Emulsions were observed to be stable against phase separation or coalescence for 120 h. 

O/W emulsions are particularly important systems for the administration of lipophilic bioactive compounds such as vitamin A and E, which are solubilized in the discontinuous phase [81]. In this work, we investigated the oxidative stability of vitamin E in the Pickering emulsions performed with ACSSNPs-CA, ACSSNPs and also with those stabilized by surfactants. HPLC analysis of vitamin E consumption was performed during an aging test under oxidative stress conditions by hydrogen peroxide, to verify chemical degradation. The results obtained from the recovery of vitamin E from the prepared emulsions showed an excellent recovery (99.5%) compared to a standard. 

**Figure 3:**
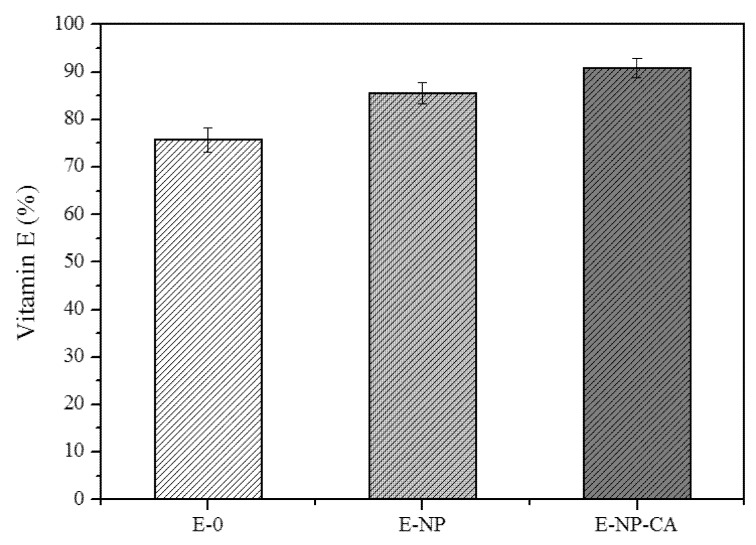
Vitamin E consumption in emulsions. Nanoparticle-free (E-0), with 1% ACSSNPs (E-NP) and with 1% ACSSNPs-CA (E-NP-CA). Each point represents mean ± SD (n = 3).

Vitamin E consumption was dependent on the type of emulsion (Figure 3). After 120 h at 40 °C, the percentage of vitamin E degradation when the emulsion was only stabilized with surfactants (E-0) was 24%. However, the degradation decreases 14.5% when 1% ACSSNPs were incorporated into this emulsion (E-NP), demonstrating the protective effect of the nanoparticle film that forms on the surface of the oil droplets. However, a greater protective effect against of vitamin E oxidation was observed when the ACSSNPs-CA were incorporated (E-NP-CA), decreasing its consumption to 9.26% of the initial value. These results clearly show the antioxidant effect of ACSSNPs-CA system.

The vitamin E degradation in emulsions under oxidative stress conditions at 40 °C for 120 h was evaluated. For this, 1.5% and 3% hydrogen peroxide was added to the emulsions. Vitamin E consumption in emulsion E-0 depends on the hydrogen peroxide concentration, decreasing 35.6% and 39% when the amount of H_2_O_2_ was 1.5% and 3%, respectively. However, there is no dependence on the amount of H_2_O_2_ in emulsions E-NP and E-NP-CA, suggesting that NPs can hinder the oxidative species diffusion from aqueous medium to the oil droplet. Since all the emulsions were prepared with the same amount of emulsifiers, the size of the oil droplets was constant, so the stability of the vitamin E is only dependent on the absence or presence of the nanoparticles in the O/W interphase. Studies show (Kargar et al.) [17,43] that silica nanoparticles reduce the oxidation rate of lipids of O/W emulsions in comparison with emulsions stabilized with Tween 20, because nanoparticles form an efficient physical barrier when they are located in the interphase of the drops, reducing the diffusion of oxidizing species. On the other hand, adding nanoparticles to emulsions increases the resistance of emulsion droplets to aggregation or coalescence, improving physical stability.

The susceptibility to oxidation of oils and lipids is a very important factor to consider in the quality, safety and efficacy of emulsions. The lipid peroxidation of emulsified lipids is a process of autoxidation through a free radical mechanism. The oxidation reaction starts when the free radicals abstract a hydrogen atom from a methylene of the fatty acid chain, the products of this reaction are mainly hydroperoxides [82,83,84,85]. In our work, the oil phase is composed of mineral oil and castor oil. The main component of castor oil is ricinoleic acid, a monounsaturated fatty acid of 18 carbons. In comparison with other oils commonly used in pharmaceutical, food and/or cosmetic emulsions, castor oil provides greater oxidative stability against peroxidation due to the presence of a hydroxyl group, beta to the double bond [86]. 

Vitamin E (α-tocopherol) is one of the best compounds in dermatological formulation for the treatment of skin aging. It is a lipid-soluble antioxidant that plays key roles in protecting cell membranes from lipid peroxidation by free radicals and in reducing photocarcinogesis [87]. Thiele et al. concluded that α-tocopherol is the major antioxidant in the human epidermis, and its depletion is an early and sensitive marker of environmental oxidative damage [88]. Vitamin E is present as the free alcohol or in its ester forms, and the beneficial effects of vitamin E-containing emulsions depend on the added concentration as well as its stability. The OH radical attacks vitamin E through different pathways: hydrogen abstraction reactions from the phenolic O-H and methyl groups and electrophilic OH addition on several positions on the aromatic ring. The most favorable pathways are the hydrogen abstraction reaction from the phenolic hydrogen and the electrophilic addition to the aromatic ring. Espinosa-García et al. proposed that the final rate constant (2.7 × 10^8^ M^−1^s^−1^ at 298 K) results from the combination of the competitive hydrogen abstraction and the addition reactions, where hydrogen abstraction represents only a 20% of the total OH radical reaction [89]. On the other hand, tocopherol can also act as an efficient quencher of singlet oxygen (^1^O_2_). Sies et al. compared values of absolute second-order rate constants for the physical quenching (k_Q_) and chemical reaction (k_r_) of α, β, γ and δ tocopherols with ^1^O_2_ in ethanol. They found that the overall rate constants (k_T_ = k_Q_ + k_r_) decrease in the order α > γ > δ > β-tocopherol [90]. Fukuzawa et al. reported that the oxidation rates of α-tocopherol by singlet oxygen depend on the photosensitizing pigment and the membrane charge of liposomes, also, the results in ethanol solutions were compared. In ethanol, using methylene blue (MB) as sensitizer, the order of physical quenching rate (k_Q_ = 3.1 × 10^8^ M^−1^s^−1^) is similar to the chemical reaction rate (k_r_ = 1.4 × 10^8^ M^−1^s^−1^) [91].

### 3.5. Color Evaluation in Pickering Emulsions with ACSSNPs-CA

**Figure 4:**
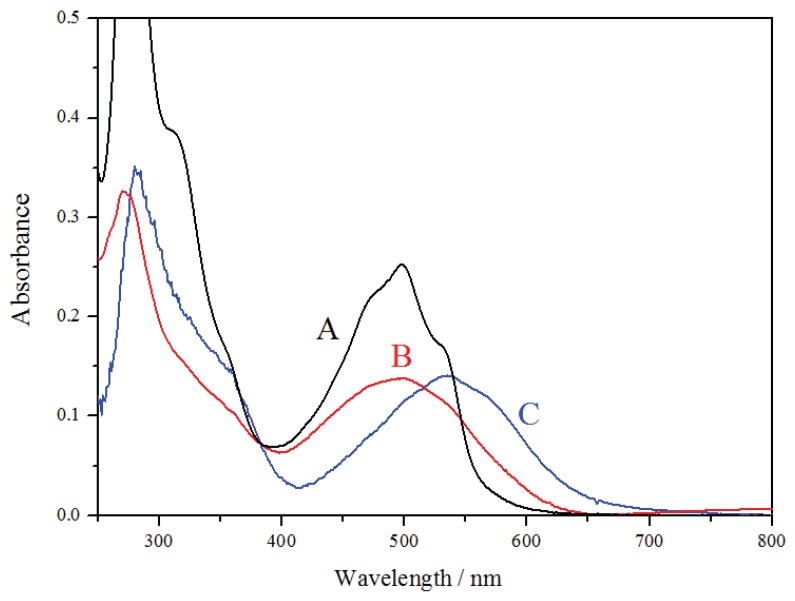
UV–vis spectra of (A) carminic acid (CA) in ethanol, (B) ACSSNPs-CA dispersed in mineral oil, and (C) ACSSNPs-CA dispersed in aqueous solution of 5% glycerin, at 35 μM.

The CA and nanosystems were dispersed in different solvents used in typical pharmaceutical formulation, and the absorption spectra (Figure 4) of CA in ethanol (A, black line) and ACSSNPs-CA dispersed in mineral oil (B, red line) and aqueous solution of 5% glycerin (C, blue line) were recorded. Typically, the absorption spectra of carminic acid in ethanol and solvents, such as water at neutral pH, show an acute peak at 278 nm corresponding to the π→π* transition and another band centered at 500 nm corresponding to the n→π* transition. Nanoparticles dispersed in nonpolar solvents, such as mineral oil, show bands similar to carminic acid in ethanol, however, when the nanoparticles are dispersed in protic polar solvents such as water or ethanol, a significant bathochromic shift with a maximum at 550 nm was observed [92]. One explanation is that the free amino groups in the shell of the nanoparticle after the amino-functionalization of bare core-shell silica nanoparticles, alkalinize the surface microenvironment, ionizing the hydroxyl groups of the attached carminic acid, producing the typical shift observed in alkaline solutions. When the carminic acid is attached onto nanoparticles, the intensity of its absorption spectrum decreases independently of the dispersion solvent.

**Figure 5:**
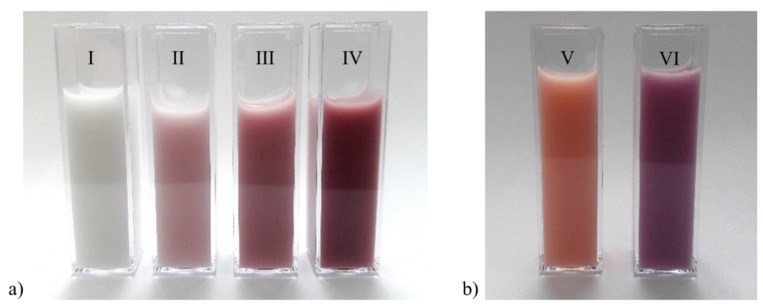
Color of emulsions with ACSSNPs and ACSSNPs-CA incorporated. (**a**) Incorporation of 1% ACSSNPs (I) and 0.1% (II), 0.5% (III) and 1% of ACSSNPs-CA (IV). (**b**) Incorporation of 1% ACSSNPs-CA adjusted to pH 3 (V) and adjusted to pH 9 (VI).

The color changes were evidenced by the addition of NPs to emulsions stabilized with surfactants (Figure 5a). The incorporation of 1% ACSSNPs does not produce changes in the color of the emulsion, however, a notorious change of color is observed by the addition of 0.1%, 0.5% and 1% of ACSSNPs-CA.

The Pickering emulsions with ACSSNPs-CA (Figure 5b) were stable immediately when the pH was adjusted to acid (pH 3) and basic (pH 9), however, a color change was observed. This change is due to the color change shown by the carminic acid linked to the surface of the nanoparticles. The carminic acid is a red pigment soluble in water in both acid and alkaline solutions and its color is very sensitive to pH, its appearance occurring at pH 7–7.7, light red to red and at basic pH of magenta. Carminic acid has an even lower coefficient of absorbance at pH 1, its pale orange color occurring at pHs below 4.5 [4,93]. 

## 4. Conclusions

A bifunctional pigment-antioxidant nanomaterial based carminic acid covalently linked onto a silica nanoparticle to confer color and improved oxidative stability to cosmetic Pickering emulsions was obtained. To achieve this purpose, a uniform dense silica core was prepared, which was then coated with mesoporous silica that was amino-functionalized and then carminic acid was attached through the action of coupling EDC/NHS agents. We obtained a spherical and homogeneous nanosystem (ACSSNPs-CA) with a diameter close to 175 nm. The results revealed that an average value of 4.30 mg of carminic acid was attached per 100 mg of nanoparticles. 

A significant change of emulsion color was observed by the addition of ACSSNPs-CA. At pH 5, samples showed an attractive pink color and when the acidity was adjusted to pH 3 and pH 9, a change in color was observed, similarly carminic acid in solution. When the colorimetric parameters and the antioxidant capacity of ACSSNPs-CA under oxidative stress were evaluated, both dropped, following a direct relation. Despite this decrease, antioxidant activity remained significant, suggesting the reuse properties of the nanomaterial.

The incorporation of nanoparticles without carminic acid notably improved the chemical stability of emulsion. However, the best protective effect against vitamin E oxidation was observed when the ACSSNPs-CA were present. A significant reduction of its consumption (only a 9.26% of the initial value), demonstrates the antioxidant effect of this new nanosystem. 

In summary, the changes in the coloration of our bifunctional pigment/antioxidant nano-excipient are a good indication of the remaining antioxidant activity of this nanomaterial and the loss of pH-dependent stability of emulsion. These easy-to-detect properties make this system a useful tool for use in formulations for food, cosmetic and/or pharmaceutical applications.
